# Predicting the Animal Susceptibility and Therapeutic Drugs to SARS-CoV-2 Based on Spike Glycoprotein Combined With ACE2

**DOI:** 10.3389/fgene.2020.575012

**Published:** 2020-10-23

**Authors:** Min Shen, Chao Liu, Run Xu, Zijing Ruan, Shiying Zhao, Huidong Zhang, Wen Wang, Xinhe Huang, Li Yang, Yong Tang, Tai Yang, Xu Jia

**Affiliations:** ^1^Non-coding RNA and Drug Discovery Key Laboratory of Sichuan Province, Chengdu Medical College, Chengdu, China; ^2^School of Life Sciences and Engineering, Southwest Jiaotong University, Chengdu, China; ^3^West China School of Public Health, Sichuan University, Chengdu, China; ^4^School of Pharmacy, Chengdu Medical College, Chengdu, China

**Keywords:** SARS-CoV-2, angiotensin-converting enzyme 2 (ACE2), nelfinavir (NFV), binding affinity [K(b)], cross-species transmission

## Abstract

Recently, a few animals have been frequently reported to have been diagnosed with severe acute respiratory syndrome coronavirus 2 (SARS-CoV-2). Whether they are SARS-CoV-2 intermediate hosts is worthy of great attention. The interaction of SARS-CoV-2 spike protein and its acceptor protein ACE2 is an important issue in determining viral host range and cross-species infection, while the binding capacity of Spike protein to ACE2 of different species is unknown. Here, we used the atomic structure model of SARS-CoV-2 and human ACE2 to assess the receptor utilization capacity of ACE2s from 10 kinds of animals. Results show that chimpanzees, domestic cats and cattles are more susceptible to infection by SARS-CoV-2. Cats in particular, such as pet cats and stray cats, interact very closely with humans, implying the necessity to carefully evaluate the risk of cats during the current COVID-19 pandemic. Furthermore, based on ACE2(cats)-SARS-CoV-2-RBD model, through high-throughput screening methods using a pool of 30,000 small molecules, eight compounds were selected for binding free energy calculations. All the eight compounds can effectively interfere with the binding of ACE2 and Spike protein, especially Nelfinavir, providing drug candidates for the treatment and prevention of SARS-CoV-2, suggesting further assessment of the anti-SARS-CoV-2 activity of these compounds in cell culture. Although we only reported the results of the simulation, and more laboratory and epidemiological investigation are required. Like cats are a risk factor, we can further detect SARS-CoV-2 according to the susceptibility of different animals, find the potential host of infection, and completely cut off the living space of the virus. Especially, cats could be a choice of animal model for screening antiviral drugs or vaccine candidates against SARS-CoV-2.

## Introduction

By June 14, 2020, the COVID-19 outbreak in China would have been well under control. SARS-CoV-2 was suddenly detected in Xinfadi seafood market in Beijing, which makes it more urgent to assess the susceptibility of animals in close contact with humans. SARS-CoV-2 has spread all over the world, and COVID-19 has posed a serious global public health emergency (Zhu et al., 2020). On one hand, the homology of SARS-CoV-2 and bat SARS-like coronavirus (bat-sl-covzc45) is higher than 85% (Boni et al., 2020; Lu et al., 2020), on the other hand, the virus is unlikely to spread directly from bats to humans due to the lack of direct contact, meaning there is an unknown intermediate host. Although bats are likely reservoir hosts for SARS-CoV-2, the identity of any intermediate host that might have facilitated transfer to humans is unknown.

Recently, pet dogs and cats have been tested to be positive for SARS-CoV-2 infection (https://finance.sina.com.cn/7x24/2020-03-19/doc-iimxxsth0356081.shtml; https://news.sina.com.cn/w/2020-03-28/doc-iimxyqwa3732342.shtml). On March 5, 2020, samples from the mouth and nose of a pet dog in Hong Kong showed SARS-CoV-2 positive but no symptom of COVID-19. The dog’s owner is a COVID-19 patient who was diagnosed in Hong Kong on February 25, 2020. Experts believe the dog was probably infected by humans, and the dog’s low level of infection with a novel coronavirus could be the first case in the world (https://finance.sina.com.cn/7x24/2020-03-19/doc-iimxxsth0356081.shtml). On March 27, 2020, Belgian public health authorities announced a pet cat in liege had been diagnosed with COVID-19. Researchers found SARS-CoV-2 in the feces of the cat, which was suffering from breathing difficulties. Belgian health officials stated subsequently the cat was infected with a novel coronavirus from its owner, and this is the first confirmed case of a pet infection in Europe (https://news.sina.com.cn/w/2020-03-28/doc-iimxyqwa3732342.shtml). Back to 2003, studies about SARS have shown that ferrets (*Mustela furo*) and domestic cats (*Felis domesticus*) were susceptible to infection by SARS-CoV and that they can efficiently transmit the virus to previously uninfected animals that are housed with them (Martina et al., 2003). Meanwhile, one laboratory study reported that cats could be infected with SARS-CoV-2 (Shi et al., 2020), and another reported that cats in Wuhan had been infected with SARS-CoV-2 during the outbreak (Zhang et al., 2020).

The specificity of the interaction between virus and its receptor is an important issue in regulating both the cross-species and human-to-human transmissions infection. SARS-CoV-2 invades cells mainly through Spike protein recognition of host cell receptor angiotensin-converting enzyme 2 (ACE2), such binding triggers a cascade events leading to the fusion between cell and viral membranes for cell entry (Ortega et al., 2020; Wan et al., 2020). ACE2 is expressed in most mammals, but not all ACE2 can be utilized by SARS-CoV-2 as the receptor (Boni et al., 2020). The utilization of ACE2 by SARS-CoV-2 can rapidly screen and narrow the range of intermediate hosts of SARS-CoV-2. Currently, it is not clear which mammals are involved in the evolution of SARS-CoV-2 and which animals are infected by SARS-CoV-2.

Like SARS-CoV, the evolutionary rate of SARS-CoV evolution were analyzed by modeling with genetic method, the results showed that SARS-CoV may spread in the bats and transmitted to other hosts such as pangolins, and have a tendency to spread across species (O’Brien et al., 2006; Le Poder, 2011; Boni et al., 2020). Objects or environments that humans and animals come into contact with can be hidden carriers of the virus. There are no clinical drugs or vaccines for SARS-CoV-2, and most experts agree that the epidemic is only under control if SARS-CoV-2 is contained in poor countries. Controlling the source of SARS-CoV-2 will therefore be a protracted battle.

In this study, we use the reported crystal structure of Spike protein binding to human ACE2 to construct 3D models of 10 kinds of animals by homologous modeling, among which 7 kinds of animals are easy to contact with humans in daily life, and the other 3 kinds of animals are commonly used model animals in the laboratory. According to the algorithm, the amino acid positions, types and sizes of the interactions between proteins were calculated, and the binding free energy was finally calculated. At the same time, high-throughput screening was used to screen out drugs that might interfere with the combination of SARS-CoV-2-RBD and ACE2 of cats. Our results may help to screen out SARS-CoV-2 intermediate hosts and to figure out the transmission model of SARS-CoV-2 and finally to control the COVID-19 disease.

## Materials and Methods

### ACE2 Sequences Alignment

The amino acid sequences of the extracellular domains of these eleven ACE2 (residues Ser19-Asp615): *Homo sapiens* (human), *Pan troglodytes* (chimpanze), *Macaca mulatta* (Rhesus monkey), *Felis catusc* (domestic cat), *Equus caballus* (horse), *Oryctolagus cuniculus* (rabbit), *Canis lupus familiaris* (dog), *Sus scrofa* (pig), *Ovis aries* (sheep), *Bos taurus* (cattle), *Mus musculus* (house mouse) were downloaded from GenBank (Supplementary File S1). Protein sequence alignments were done using Clustal Omega.

### Protein–Protein Docking

Homology model of the target ACE2 protein was built by modeller9.18 using crystal structure of human ACE2 (PDB: 1R42) as template. Hundred independent structures were constructed and the one with best DOPE score was chosen for further energy minimization in Amber18 using ff14SB force field. Rosetta3.7 was used to perform protein-protein docking to get the ACE2-SARS-CoV-2-RBD complex, and default values are used for parameter setting. The PDB of the SARS-CoV-2 spike protein is 6VSB (Lan et al., 2020).

### Molecular Dynamics Simulation

The ACE2-SARS-CoV-2-RBD complex was immersed in an octahedron box of TIP3P water that was extended by 10 Å from the solute. In the simulation system, the amount of Na + is exactly equal to the amount of negative charge, so as to neutralize the system charge and make the total charge zero. Amber ff14SB force field was used to parameterize the protein. 10,000 steps of minimization with constraints (10 kcal/mol/Å2) on heavy atoms of complex, including 5,000 steps of steepest descent minimization and 5,000 steps of conjugate gradient minimization, was used to optimize each system. Then each system was heated to 300 K within 50 ps followed by 50 ps equilibration in NPT ensemble. Finally, 100 ns MD simulation on each system at 300 K was performed. The minimization, heating and equilibrium are performed with sander program in Amber18. The 100 ns production run was performed with pmemd.cuda. Based on the 100 ns MD simulation trajectory, binding free energy (ΔG) between spike and viral ACE2 receptor was calculated with MM/GBSA method (The MMGBsa.py module in the Amber program was used for the MM/GBSA calculations)according to the following equation (Genheden and Ryde, 2015):Δ*G* = Δ*H* – *T*Δ*S* = Δ*E*_*ele*_ + Δ*E*_*VDW*_ + Δ*G*_*g*__*b*_ + Δ*G*_*np*_ – *T*Δ*S*. Where ΔEele and ΔEVDW refer to electrostatic and van der Waals energy terms, respectively. ΔGgb and ΔGnp refer to polar and non-polar solvation free energies, respectively. Conformational entropy (TΔS) was not calculated for saving time. Besides, the ligands were compared based on the same target, so it is reasonable to ignore the entropy.$\sigma =\sqrt{\frac{1}{N}\,\sum_{I=1}^{N}{\left(x_{i}-u\right)}^{2}}$, SD was calculated according this equation where x_*i*_ represents the binding free energy in each frame.

### Construction of Small Molecular Ligands

Each sub-library (FDA, investigational-only, world-not-FDA^1^) was downloaded from the zinc database. The 2D structure of the compound was then converted into the corresponding 3D coordinates using the Babel server^2^. Then the model was converted to pdbqt format by prepare_receptor4.py script (From the AutodockTool package)with assigning atomic types and atomic charges. All rotatable bonds in the molecule are set to be flexible for flexible docking. Vina1.1.2 was used for molecular docking.

### Binding Free Energy Calculation Between ACE2(cats)-SARS-CoV-2-RBD and Small Molecular Ligand

The ACE2-SARS-CoV-2-RBD complex structure of cats that obtained from Section “Protein–Protein Docking” was used for docking with small molecular ligand to calculate binding free energy. The minimum conformation of the docking was used as the initial position of the drug molecule.

Each simulation system was immersed in a cubic box of TIP3P water with 10 Å distance from the solute. The Na^+^ or Cl^–^ was applied to neutralize the system. General Amber force field (GAFF) 15 and Amber ff14SB force field were used to parameterize the ligand and protein respectively. 10,000 steps of minimization with constraints (10 kcal/mol/Å2) on heavy atoms of complex, including 5,000 steps of steepest descent minimization and 5,000 steps of conjugate gradient minimization, was used to optimize each system. Then each system was heated to 300 K within 0.2 ns followed by 0.1 ns equilibration in NPT ensemble. Finally, 5 ns MD simulation on each system at 300 K was performed. The minimization, heating and equilibrium are performed with sander program in Amber18. The 5 ns production run was performed with pmemd.cuda. Based on the 5 ns MD simulation trajectory, binding free energy (ΔG) was calculated with MM/GBSA method according to the following equation: ΔG_*cal*_ = ΔH-TΔS = ΔE_*vdw*_ + ΔE_*ele*_ + ΔG_*gb*_ + ΔG_*np*_-TΔS, where ΔE_*ele*_ and ΔE_*vdw*_ refer to electrostatic and van der Waals energy terms respectively. ΔG_*gb*_ and ΔG_*np*_ refer to polar and non-polar solvation free energies respectively. Conformational entropy (TΔS) was not calculated for saving time. Besides, the ligands were compared based on the same target, so it is reasonable to ignore the entropy.

## Results and Discussion

### Comparison of Amino Acid Sequences of ACE2 Among 10 Kinds of Animals

In order to analyze the possibility of SARS-CoV-2 infection on mammals that humans may come into contact with in daily life, we collected the amino acid sequence of ACE2 of pet cats, dogs, livestock cattle, horses, sheep and pigs, as well as laboratory animals Rhesus monkey, chimpanzees, mice and rabbits in Genbank. We aligned the amino acid sequence of the extracellular domains of these eleven ACE2, the results are shown in Table 1. Compared with the ACE2 of human, the homology of amino acid sequence was between 95.0%∼67.4%. It’s worth noting that, as a commonly used model animal in the laboratory, mice had the lowest homology. That is to say, the N-terminal peptidase domain of ACE2 in these animals is very similar to that of the known human hosts of infection. Chimpanzees and Rhesus monkey seem to be the most susceptible to SARS-CoV-2, while mice is the least sensitive.

**TABLE 1:** Comparison of amino acids homology of ACE2 between human and other organisms.

**Species**	**Protein-ID**	**Identity%**
*Homo sapiens* (human)	XP_011543853.1	100
*Pan troglodytes* (chimpanzee)	XP_016798468.1	95.0
*Macaca mulatta* (Rhesus monkey)	XP_028697658.1	91.2
*Equus caballus* (horse)	XP_001490241.1	83.1
*Oryctolagus cuniculus* (rabbit)	XP_002719891.1	82.5
*Felis catus* (domestic cat)	XP_023104564.1	81.2
*Canis lupus familiaris* (dog)	NP_001158732.1	80.1
*Sus scrofa* (pig)	XP_020935033.1	78.8
*Ovis aries* (sheep)	XP_011961657.1	78.4
*Bos taurus* (cattle)	XP_024843618.1	77.3
*Mus musculus* (mouse)	NP_001123985.1	67.4

### Binding Affinity (ΔG) Values of the Interaction Between Spike and Viral ACE2 Receptor

To further analyze the possibility of infection in these animals, we determined the crystal structure of the SARS-CoV-2 spike receptor-binding domain (RBD) bound with the cell receptor ACE2, calculated the binding free energy. The predicted results are shown in Table 2, chimpanzees have the highest binding affinity, even higher than human, while gradually decreases in order of cats, cattle, Rhesus monkey, dogs, pigs, horses, sheep, mice, and rabbits. Although they belong to different species, they all have the binding affinity of the interaction. The binding affinity of cats and chimpanzees are very similar to human, while rabbits and mice are the lowest. Higher affinity values might be related to the dynamic of infection and the rapid spread observed for this virus. These data suggested that the higher binding affinity of RBD of coronavirus to ACE2 will confer the virus higher infectivity and pathogenicity (Ortega et al., 2020; Xu et al., 2020).

**TABLE 2:** Binding affinity (ΔG) predicted values for the interaction between spike and viral ACE2 receptor.

**Protein-protein complex (spike/Viral ACE2)**	**ΔG (kcal/mol)**	***SD***
Chimpanzee	−50.7659	±11.7683
Human	−46.7995	±14.6343
Cat	−44.8766	±4.7180
Cattle	−40.281	±5.5411
Rhesus monkey	−39.326	±5.2482
Dog	−37.7814	±7.2897
Pig	−30.719	±6.5672
Horse	−28.4732	±7.4204
Sheep	−26.3634	±11.9568
Mouse	−21.9914	±6.9997
Rabbit	−16.749	±7.5223

**FIGURE 1:**
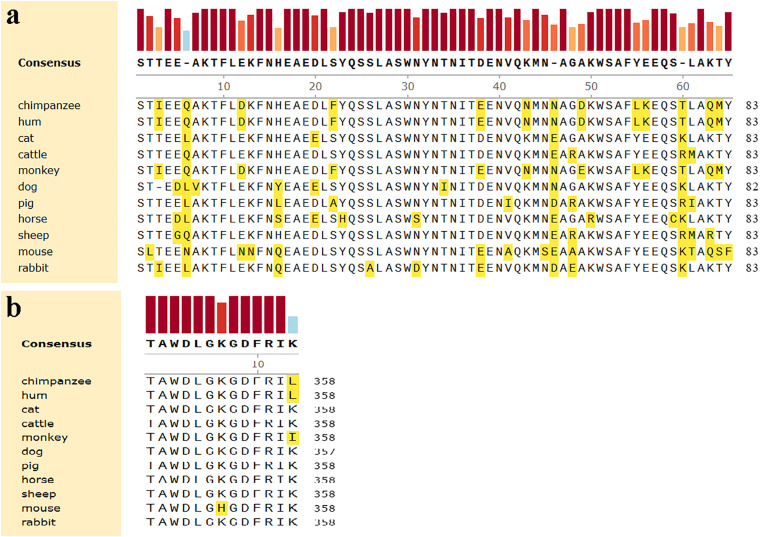
Comparison of amino acids homology of ACE2 in the helical structure and the folding between human and other organisms. **(a)** The helical structure of 19–83 aa. **(b)** The folding structure of 347–358 aa. The different amino acids are shown in yellow.

**FIGURE 2:**
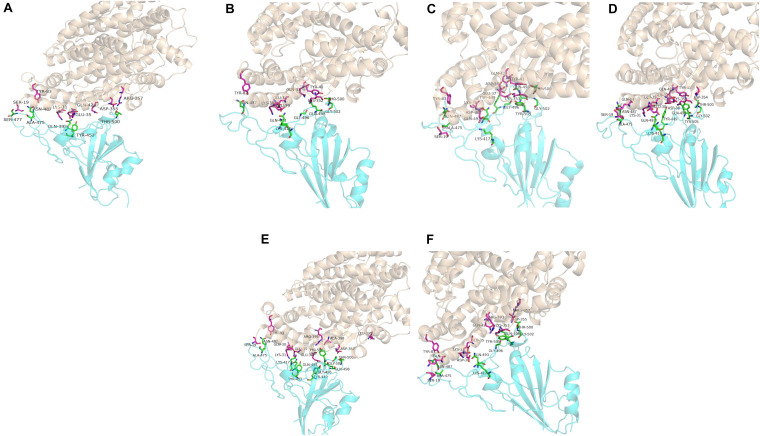
Overall structure of SARS-CoV-2 RBD bound with ACE2. **(A–F)** Are respectively human, dogs, chimpanzees, cattles, cats, Rhesus monkey. ACE2 is colored light coffee. SARS-CoV-2 RBD core is colored cyan. Amino acid interaction sites are also shown.

Computer modeling of interaction between SARS-CoV-2 RBD and ACE2 has identified some residues potentially involved in the actual interaction. Spike protein contacts with the helical structure of 19-83aa of human ACE2 and the folding structure which in the 347-358aa region of human ACE2, producing intermolecular interactions. The results are shown in Table 3 and Figure 1. Structural analysis revealed a total of 11 residues of the SARS-CoV-2 RBD contact 13 residues of the human ACE2, and there are 15 hydrogen bonds at the SARS-CoV-2 RBD/ACE2 interface. It is found that cat and human ACE2 are very similar when it binds to RBD, a total of 11 residues of the RBD contact 12 residues of the ACE2, 14 hydrogen bonds at the SARS-CoV-2 RBD/ACE2 interface. Structural analysis revealed that most of the cats-critical ACE2 binding residues in ACE2-SARS-CoV-2-RBD had highly conserved or side chain characteristics similar to those in humans (Table 3 and Figure 2). Taken together, these results show that the SARS-CoV-2 RBD/ACE2 of cats interfaces share substantial similarity in the number of interacting residues, and hydrophilic interaction networks. In the study of SARS, the infection experiments on many kinds of animals show that ferrets and domestic cats can be used as potential animal infection models such as vaccine and drug screening (Martina et al., 2003). This is consistent with the results of recent virus infection experiments: ferrets and cats have effective replication ability. Virus RNA was found in the nose, soft jaw, tonsil and small intestine of ferrets, but no virus was detected in other organs, which proved that SARS-CoV-2 was only replicated in the upper respiratory tract (Chen, 2020). A research in human body have been reported that: ACE2 receptor is highly expressed in human nasal cells, but not detected in lung cells (Wu et al., 2020). Meanwhile, the expression of ACE2 in the small intestine is high, which is consistent with the recently reported gastrointestinal tract as a potential route of SARS-CoV-2 infection (Lu et al., 2020; Wang et al., 2020). Compared with human, ACE2 and SARS-CoV-2 binding affinity of mice are very low, similar interacting residuals were fewer, and hydrophobic interaction networks are weaker. Therefore, SARS-CoV-2 may replicate inefficiently in mice and rats, ruling them out as animal models to test vaccine or antiviral drugs candidates against SARS-CoV-2.

**TABLE 3:** Potential interaction between the S protein receptor binding domain (RBD) and ACE2 of a variety of animals.

**RBD**	**Human**	**Chimpanzee**	**Cat**	**Cattle**	**Rhesus monkey**	**Dog**	**Pig**	**Horse**	**Sheep**	**Mouse**	**Rabbit**
405D	−	−	−	−	−	−	−	−	−	387R	−
417K	30D	30D	30E	30E	30D	29E	30E	30E	30E	−	Q30
449Y	38D	38D	38E	38D	−	−	−	38E	38D	38D	−
453Y	34H	−	34H	−	−	−	−	−	−	−	−
475A	19S	19S	19S	19S	19S	−	19S	19S	19S	−	−
477S	19S	−	−	−	−	−	−	−	−	−	−
487N	24Q	−	−	24Q	24Q	−	−	−	−	24N	−
487N	83Y	83Y	83Y	−	83Y	82Y	83Y	83Y	−	−	83Y
493Q	31K	31K	31K	31K	31K	30K	31K	31K	−	−	31K
493Q	35E	−	35E	35E	−	34E	35E	35E	−	34Q	35E
495Q	−	−	−	−	−	−	−	−	31K	−	−
496G	−	353K	355K	−	353K	352K	−	353K	352K	−	−
498Q	42Q	42Q	38E	−	42Q	41Q	−	−	−	−	−
498Q	353K	353K	355K	352K	353K	352K	−	353K	−	−	−
500T	−	41Y	−	41Y	−	40Y	41Y	−	−	−	−
500T	355D	−	−	354D	355D	−	355D	355D	−	355D	−
500T	357R	−	357D	−	357R	−	−	−	−	−	−
502G	353K	353K	355K	352K	353K	352K	353K	353K	352K	353H	353K
505Y	−	−	395R	−	−	−	−	−	−	−	−
505Y	37E	37E	388A	37E	393R	−	−	386A	−	386A	−

### Docking Results of 7496 Drugs Against ACE2(Cats)-SARS-CoV-2-RBD Model

The 7,496 drugs obtained from the zinc database were screened for molecular docking. Among them, 57 of the 2,100 compounds approved by the FDA have a docking score better than −8.0 kcal/mol. There were 4,264 compounds approved by regulatory agencies other than the FDA. Among them, there were 124 compounds with the docking score better than −8.0 kcal/mol. 67 of the 1132 compounds in clinical trials have a docking score better than −8.0 kcal/mol. Further, eight top compounds showed the docking score in a range of −8.1 to −9.9 kcal/mol were selected from docking results of homology model (Table 4).

**TABLE 4:** Eight drugs selected from the ACE2(cats)-SARS-CoV-2-RBD model.

**Drug name**	**ID**	**Data**	**Affinity (kcal/mol)**
Dihydroergotoxine	ZINC14880002	World-not- FDA	−9.9
Ergotamine	ZINC52955754	FDA	−9.2
Tegobuvir	ZINC100057121	Investigational-only	−9.0
Fiduxosin	ZINC29747110	Investigational-only	−9.0
Dihydroergotamine	ZINC3978005	FDA	−9.0
Saquinavir	ZINC26664090	FDA	−8.5
Nelfinavir	ZINC3833846	FDA	−8.4
Setrobuvir	ZINC100341584	Investigational-only	−8.1

### Docking Results of Eight Drugs Against ACE2(Cats)-SARS-CoV-2-RBD Model

So far, only Remdesivir has been approved for the treatment of COVID-19 in Japan, and in the world no corresponding vaccine has been approved. For these confirmed cases, they need antiviral drugs or other drugs to treat SARS-CoV-2 infection. More potentiality compounds need to be tested. In the presence of ACE2-RBD complex formation, that is to say, when symptoms of infection may already be present, the interference of small molecules with ACE2(cats)-SARS-CoV-2-RBD complex can be assessed by assessing the likelihood of small molecules entering the lumen of the complex. In order to reduce the cost of compounds screening, eight compounds were selected based on virtual screening and docking scores and its showed as Table 5.

**TABLE 5:** The calculated binding energies of ligand to ACE2(cats)-SARS-CoV-2-RBD.

Energy*	**Nelfinavir**	**Saquinavir**	**Fiduxosin**	**Dihydroergotoxine**	**Ergotamine**	**Dihydroergotamine**	**Tegobuvir**	**Setrobuvir**
ΔEvdw	−52.85613.7235	−63.50743.5899	−60.59374.4183	−55.37652.9996	−53.56603.0186	−43.74595.0458	−45.36063.3071	−49.82222.8510
ΔEele	−47.189810.4863	−41.32943.5899	−36.33259.3597	−49.13675.1493	−48.47846.7194	−24.68318.3207	−23.92643.6684	−33.583010.0571
ΔGgb	56.506010.2482	66.12004.1245	58.50356.4840	67.87303.8831	74.02844.9328	44.52496.6776	46.12893.7154	5.62798.6629
ΔGnp	−7.25020.4905	−8.86630.2953	−7.46100.3074	−6.77050.2448	−6.89820.1723	−5.84340.5785	−6.36490.3356	−6.76130.2702
ΔGcal	−50.79014.1892	−47.58313.4446	−45.88374.7208	−43.41073.1456	−34.91423.5143	−29.74756.9375	−29.52313.0950	−17.37264.1140

*ΔE_*vdw*_, van der Waals energy terms; ΔE_*ele*_, electrostatic energy; ΔG_*gb*_, polar solvation free energy; ΔGnp, non-polar solvation free energy; ΔGcal, final estimated binding free energy calculated from the above terms (kCal/mol). The numbers after the “±” sign are Standard deviations.

Nelfinavir is a clinically important antiviral drug, which can inhibit the production of human immunodeficiency virus (HIV) and it can inhibits the Herpes Simples Virus 1(HSV-1) (Kalu et al., 2014; Gantt et al., 2015). Our docking results show that five hydrogen bonds, including GLU-30, ASN-33, PRO-391 and LYS-417, are maintained on the binding of nelfinavir with the ACE2 and Spike glycoprotein complex. Among them, GLU-30 and nelfinavir maintain two hydrogen bonds. In the previous studies, some scientists have demonstrated the antiviral effect of nefinavir using Vero cell lines infected with SARS CoV (Yamamoto et al., 2004; Hsieh et al., 2010).

Saquinavir is the first protease inhibitor used to treat patients with HIV infection (Figgitt and Plosker, 2000). One hydrogen bonds involving THR-27 and van der Waals energy maintained upon the binding of saquinavir with ACE2 and Spike glycoprotein complex interface. Previous studies shown that saquinavir could inhaibit the RNA-dependent RNA polymerase (RdRP) activity (Beck et al., 2020).

Tegobuvir and Setrobuvir, Non-Nucleoside Analog inhibitors of the Hepatitis C virus(HCV) (Zeuzem et al., 2012; Mallalieu et al., 2014; Wyles et al., 2014). Tegobuvir, the hydrogen bond involving ARG-403 maintained upon binding of tegobuvir with ACE2 and Spike glycoprotein complex interface, with additionally van der Waals energy, but for Setrobuvir, it was mainly combined with ACE2 and Spike glycoprotein complex interface through van der Waals potential energy.

**FIGURE 3:**
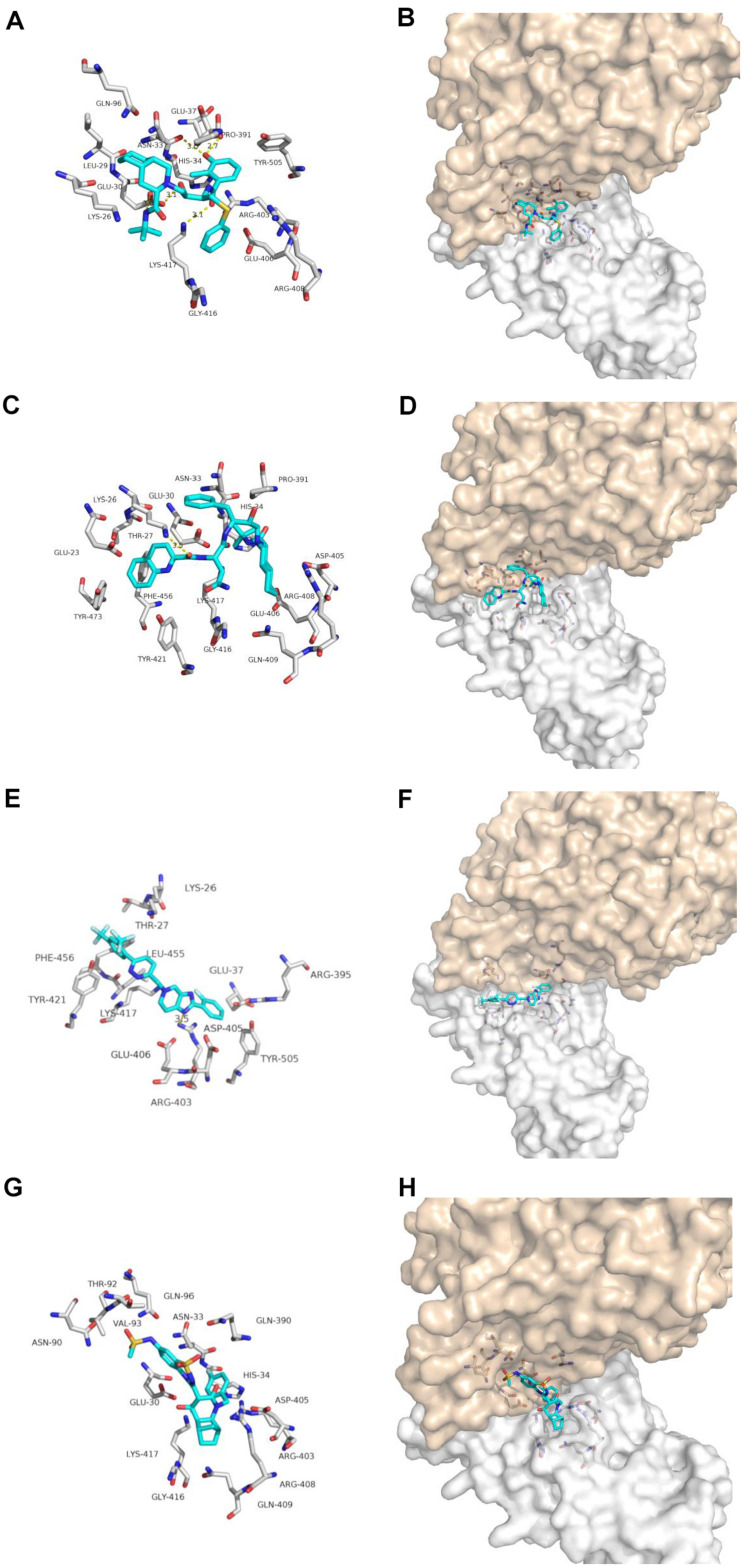
Binding model of antiviral drugs with ACE2 and Spike glycoprotein complex interface. Antiviral drug binding model with ACE2 and Spike glycoprotein complex interface. **(A,C,E,G)** are the binding models of ACE2 and Spike glycoprotein complexes with antiviral drugs, Olysio (cyan) and related residues (off-white), **(B,D,F,H)** are compound Olysio (blue). The interaction-green model is located at the interface of the Spike glycoprotein complex (between coffee and white surface). The yellow dotted line indicates the interaction distance (A). **(A,C,E,G)** or **(B,D,F,H)** are nelfinavir, saquinavir, tegobuvir, and setrobuvir, respectively.

Our simulation results show that these four antiviral drugs can enter the interface of ACE2 and Spike glycoprotein complex, and they all have antiviral activity and can treat viral infections (Figure 3). Therefore, we speculate that these four drugs have potential activity for the treatment of COVID-19. But this conclusion needs to be needs to be further verified by *in vivo* experiments.

**FIGURE 4:**
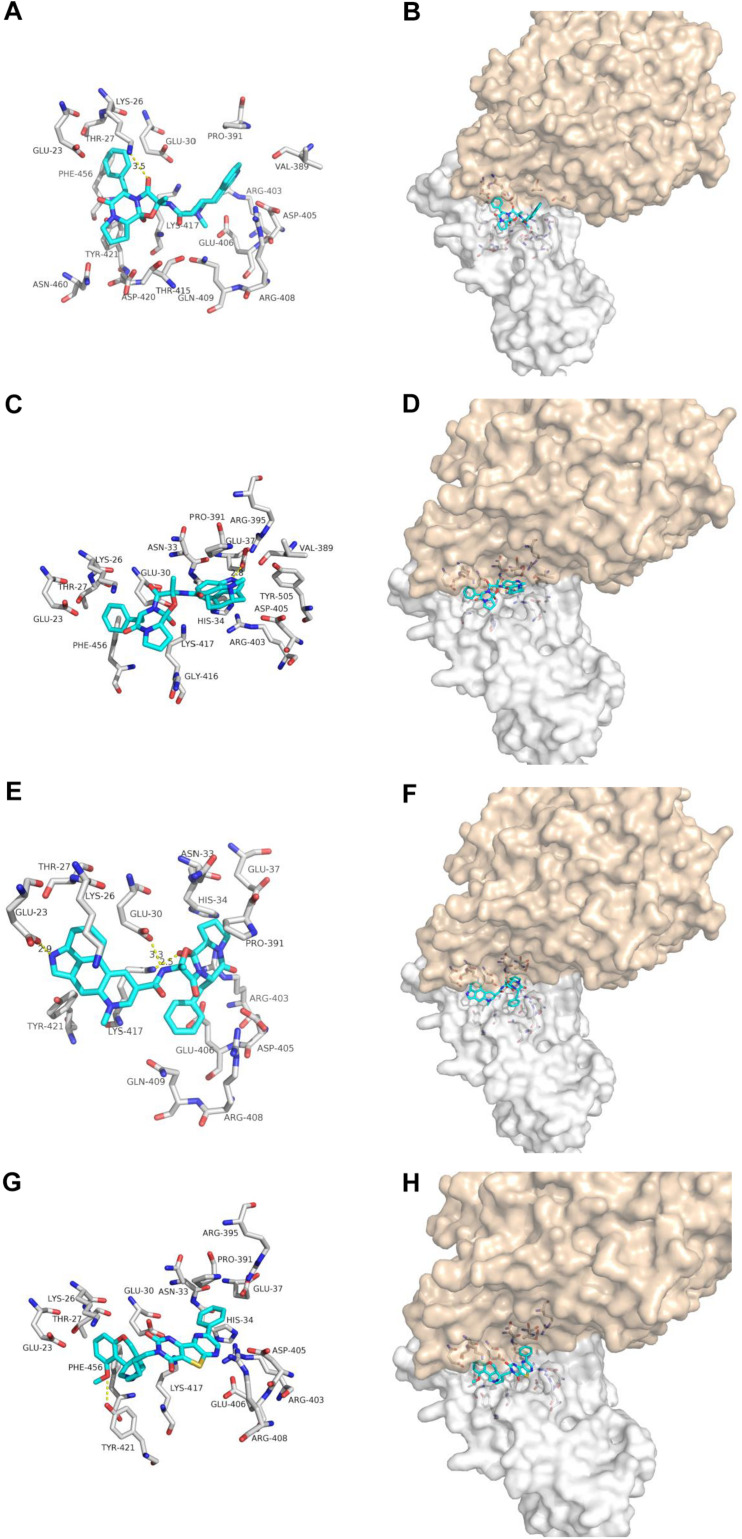
The binding model of non-antiviral drugs with the interface of ACE2 and Spike glycoprotein complexes. Antiviral drug binding model with ACE2 and Spike glycoprotein complexes interface. **(A,C,E,G)** are the binding models of ACE2 and Spike glycoprotein complexes with antiviral drugs, Olysio (cyan) and related residues (off-white), **(B,D,F,H)** are compound Olysio (blue). The interaction-green model is located at the interface of the Spike glycoprotein complex (between coffee and white surface). The yellow dotted line indicates the interaction distance (A). **(A,C,E,G)** or **(B,D,F,H)** are dihydroergotamine, dihydroergotoxine, ergotamine, fiduxosin, respectively.

Dihydroergotamine, Dihydroergotoxine and Ergotamine are the synthetic drugs developed in the 20th century for treating migraine (Metra et al., 1995; Villamil-Hernandez et al., 2014; Connelly et al., 2020) (Figure 4). Our docking results show that one hydrogen bonds involving LYS-26 and van der Waals energy maintained upon the binding of DHE with the interface of ACE2 and Spike glycoprotein complex. The interface between DDE and ACE2 and Spike glycoprotein complex maintains a hydrogen bond involving GLU-37 and Van der Waals energy. As for ergotamine, when ergotamine binds to the ACE2 and Spike glycoprotein complex, three hydrogen bonds involving GLU-23, GLU-30 and LYS-417 are maintained. Our simulation results further confirmed that DHE, DDE and ERG can bind to the interface of ACE2 and spike glycoprotein complex.

Fiduxosin, one of the α_1_-Adrenocepter Antagonists, is used to treat the lower urinary tract symptoms (LUTS) (Brune et al., 2002; Hancock et al., 2002) (Figure 4). Our ducking results showed that one hydrogen bonds involving THR-421 and van der Waals energy maintained upon the binding of fiduxosin with the interface of ACE2 and Spike glycoprotein complex. Thus making them as candidates for further *in vitro* evaluation of anti-SARS-CoV-2 activity.

### Binding Free Energy Calculated by MM/GBSA

Through the simulation trajectory of 100 ns molecular dynamics simulations, we calculated the binding free energy of five drugs by MM/GBSA methods. The bonding free energy for the interface of ACE2 and spike glycoprotein of eight compounds were calculated. Nelfinavir has the strongest binding free energy, suggesting it can be tested their anti-SARS-CoV-2 infection *in vitro* (Table 5).

## Conclusion

Based on the potential interaction between S protein and mammalian ACE2, it was speculated that SARS-CoV-2 preserved the ability to infect many mammals including chimpanzees, cats, cattle and Rhesus monkey. Cats’ binding free energy, as well as key amino acids, are highly similar to humans, meaning they could serve as model animals for developing vaccines and drugs, implicating these animal species as possible intermediate hosts or animal models for SARS-CoV-2 infections. About 60 percent of people infected with the virus are asymptomatic carriers and bats are a special case of SARS asymptomatic carriers, so these animals can carry the virus and infect people (Case Study Shows Asymptomatic Transmission of COVID-19 in China; Kupferschmidt, 2020). Modeling with genetic method, according to the HcoV-OC43 and MERS-CoV the evolutionary rate of SARS-CoV evolution are analyzed, the results showed that SARS-CoV may spread in the bats is transmitted to other hosts such as pangolins, dozens of years later it was the third time in the 17 years of coronary virus outbreak, will probably have a virus across species boundaries (O’Brien et al., 2006; Le Poder, 2011; Boni et al., 2020). Considering the widespread of stray cats, wildlife markets and stock farms in Wuhan, it was not strange that these animals could serve as potential intermediate hosts of SARS-CoV-2. Therefore, pets exposed to the patient should be screened for SARS-CoV-2. During the process of epidemic prevention, we should prevent the predict possible of zoonosis event or cross-infection in the future. However, these are still preliminary results predicted by sequence analysis, and more laboratory and epidemiological investigation are required.

## Data Availability Statement

The raw data supporting the conclusions of this article will be made available by the authors, without undue reservation.

## Author Contributions

MS, YT, and XJ designed the study. MS, CL, and RX performed most studies, but a few were carried out by ZR, SZ, HZ, WW, XH, and LY. All authors contributed thoughts and advice. MS and CL analyzed and interpreted the data. MS wrote the text, and the other authors contributed to the final text presentation. All authors have approved the submission.

## Conflict of Interest

The authors declare that the research was conducted in the absence of any commercial or financial relationships that could be construed as a potential conflict of interest.

## Footnotes

1 http://zinc.docking.org/

2 http://openbabel.sf.net
